# Small family, big impact: RNL helper NLRs and their importance in plant innate immunity

**DOI:** 10.1371/journal.ppat.1011315

**Published:** 2023-04-20

**Authors:** Svenja C. Saile, Farid El Kasmi

**Affiliations:** Center for Plant Molecular Biology, Eberhard Karls University of Tübingen, Tübingen, Germany; Shanghai Center for Plant Stress Biology, CHINA

Plants evolved a sophisticated, receptor-based, innate immune system. Cell surface localized pattern recognition (PRR) and intracellular nucleotide-binding leucine-rich repeat (NLR) receptors detect pathogen-associated molecular patterns or pathogen-derived effector molecules, respectively, and induce a range of common immune responses. These include Ca^2+^ fluxes, reactive oxygen species production, and mitogen-activated protein kinase activation [1]. Recent studies have demonstrated an interdependency and mutual potentiation of the 2 receptor systems [2,3]. Based on their N-terminal domains and their phylogeny, NLRs are classified in coiled-coil (CC) domain, Toll-like/interleukin-1 receptor resistance (TIR) domain, and RESISTANCE TO POWDERY MILDEW 8-like CC (CC_R_) domain containing NLRs, referred to as CNLs, TNLs, and RNLs, respectively [4]. In *Arabidopsis thaliana* (hereafter *Arabidopsis*), multiple PRRs and effector sensing NLRs (some CNLs and all tested TNLs) require the presence of RNLs, also termed helper NLRs, to activate full immunity [5,6]. RNLs form a small and evolutionary conserved clade comprised of 2 subfamilies, the *ACTIVATED DISEASE RESISTANCE 1* (*ADR1*) and *N REQUIREMENT GENE 1* (*NRG1*) families that have separated before the divergence of angiosperms [4]. The *Arabidopsis* genome bares 3 *ADR1* and 2 *NRG1* full-length genes required for full immunity [7–9]. Although RNLs represent only a relatively small part of the NLR gene repertoire in most angiosperms [4,10], they are of outmost importance for plants to fight off invading pathogens. Here, we highlight recent findings of how RNLs function during immunity and discuss mechanisms of RNL activation.

## RNLs are central nodes in the plant immune receptor network

In *Arabidopsis*, ADR1 and its 2 paralogs ADR1-LIKE 1 and ADR1-LIKE 2 act redundantly downstream of multiple CNLs and TNLs [7,9,11,12], are required for immune signaling induced at the cell surface by PRRs (see below [5,6]) and basal immunity [7]. *Arabidopsis* NRG1.1 and NRG1.2 serve as redundant signaling components specifically required for TNL-induced immunity and contribute to basal resistance in the absence of ADR1s [8,9]. Interestingly, functional redundancy has not only been reported within the RNL subfamilies, but also between both subfamilies [11,13]. It is remarkable, however, that in *Arabidopsis* and *Nicotiana benthamiana* ADR1s and NRG1s contribute to some sensor NLR-triggered immune responses in an unequally redundant manner. In *Arabidopsis*, the ADR1 subfamily is predominantly involved in mediating resistance (including transcriptional reprogramming) and the NRG1s in triggering cell death [11]. In contrast, in *N*. *benthamiana* NRG1 is sufficient to mediate resistance against *tobacco mosaic virus* (trigger of the TNL N) or *Pseudomonas syringeae* (trigger of the TNL Roq1) infections, since knocking out *NbADR1* does not affect these resistance responses [13–16]. This may indicate that during evolution, the RNL subfamilies have subfunctionalized in different species or that the preference of the upstream TNLs (see below) for one or the other subfamily has changed. It will be interesting to see whether a similar subfunctionalization can also be observed in other angiosperm species.

Why did plants evolve 2 RNL subfamilies that act more or less redundantly? RNLs represent downstream signaling hubs of at least 2 pathogen-sensing receptor networks. Consequently, genetic loss of RNLs or pathogen-mediated disturbance of their function would impair immune signaling by multiple immune receptors and thus, lead to enhanced disease susceptibility against various pathogens [11,15]. Plants must withstand rapidly evolving pathogens and need to evade suppression by effector proteins. This can be achieved by evolving and employing redundant RNLs that form robust immune signaling nodes. As pathogens require susceptible hosts for colonization, RNLs represent ideal targets for effector proteins. However, no effectors that specifically target RNLs have been identified yet.

## RNLs form distinct signaling modules with EDS1 family members

The genetic requirement of RNLs by TNLs is well documented. Until recently, it was not clear how exactly effector recognition by TNLs leads to RNL activation. Immune induction by most TNLs depends on the presence of genes encoding for the structurally unique plant-specific lipase-like proteins ENHANCED DISEASE SUSCEPTIBILITY 1 (EDS1), PHYTOALEXIN DEFICIENT 4 (PAD4), and SENESCENCE-ASSOCIATED GENE 101 (SAG101) [17]. EDS1 forms exclusive heterodimers with either PAD4 or SAG101 to regulate distinct immune outputs that are reminiscent of RNL-mediated immune responses [18]. Genetic and biochemical data demonstrated that EDS1-PAD4 function together with the ADR1s during basal resistance and TNL- as well as some CNL-induced immune responses [19]. In contrast to the EDS1-PAD4 heterodimer, EDS1-SAG101 act specifically in concert with the NRG1 subfamily during TNL-induced immunity [12,19,20]. Phylogenetic analysis suggests that the EDS1 family evolved after the emergence of NLRs but before the divergence of RNLs into 2 subfamilies, potentially to function with RNLs to mediate immune responses downstream of TNLs. Indeed, EDS1 heterodimers were shown to connect TNL effector recognition and RNL activation to a full immune response [19,21,22].

**Fig 1:**
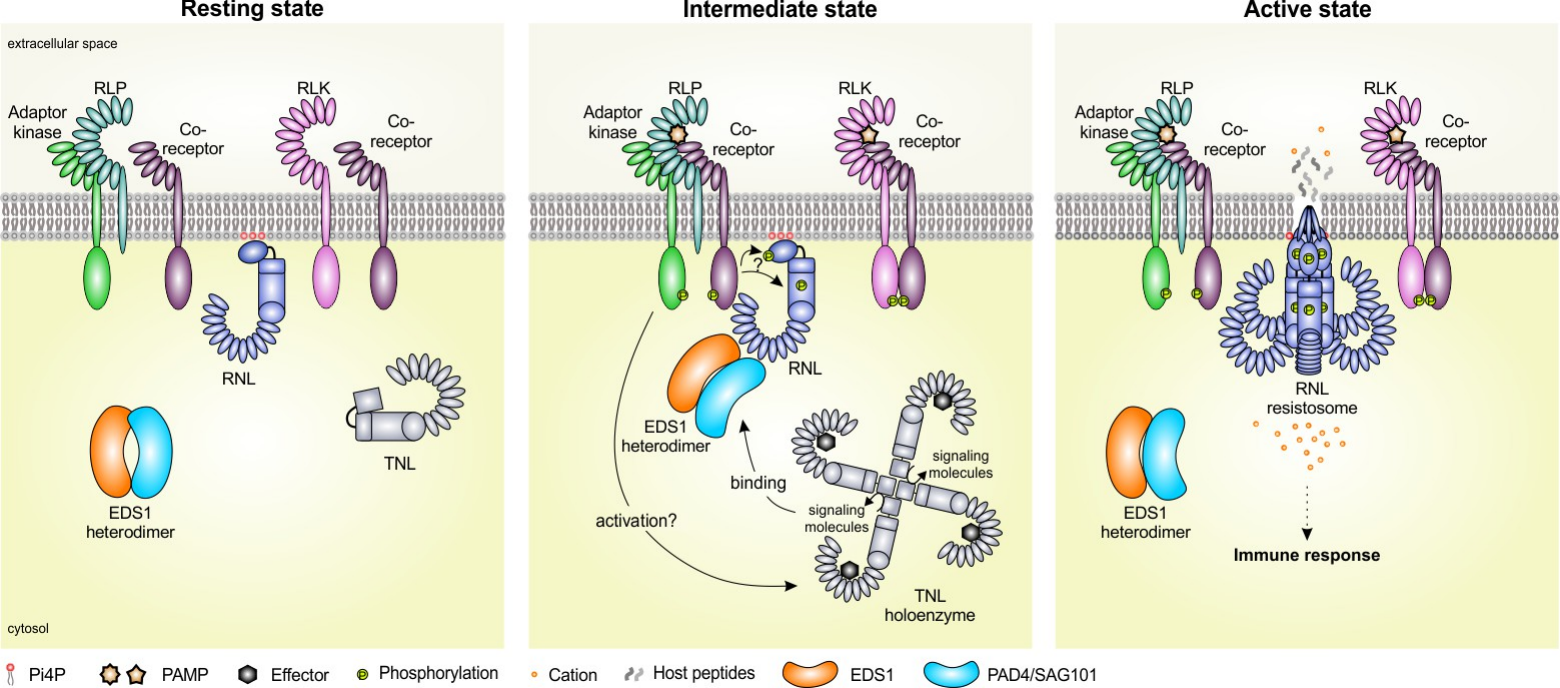
Schematic model of RNL activation and function during *Arabidopsis* innate immunity. ** Resting state:** In the absence of pathogens, RNLs exist likely as inactive monomers and in close proximity to cell surface-localized PRRs (including RLPs and RLKs). RNLs localize at the PM via direct interaction of their CC_R_ domains with the anionic phospholipid Pi4P. **Intermediate state:** The perception of PAMPs by PRRs leads to the recruitment of a co-receptor and activates immune signaling by auto- and transphosphorylation. Components of the activated PRR core-complex may phosphorylate RNLs and “prime” them prior to full activation. Components of the activated PRR core-complex may also associate and activate downstream TNLs. TNLs, however, are also activated by recognition of pathogen-derived effector proteins. Activated TNLs assemble into a tetrameric complex. TIR domains have enzymatic activity and produce small signaling molecules that can bind directly to EDS1 heterodimers. Binding of these signaling molelcules to the EDS1-PAD4 heterodimer was shown to cause a conformational change in PAD4 that promotes the interaction with RNLs and may drive their full activation. **Active state:** Activation of RNLs may lead to the exposure of their N-terminal α1-helix that could trigger the dissociation of the EDS1 heterodimer and the oligomerization of RNLs into a PM-associated resistosome. Activated RNLs promote cation influx (directly or indirectly) that leads to immune responses. It may also be possible that activated RNLs are involved in the release of host peptides/molecules that could activate immune signaling in neighboring cells. PAMP, pathogen-associated molecular pattern; PM, plasma membrane; PRR, pattern-recognition receptor; RLP, receptor-like protein; TIR, Toll-like/interleukin-1 receptor resistance.

Following effector recognition by TNLs, the TIR domain embedded enzymatic NADase and ADPR polymerase-like activity produces small signaling molecules that initiate a physical association of EDS1-PAD4 with ADR1 family members and EDS1-SAG101 with NRG1s [19,21,23–26]. This association is thought to be required for RNL activation. However, very recent data suggest that EDS1-SAG101 is absent in NRG1.1 high-molecular weight complexes (potentially resistosomes) [27]. This suggests that, once activated, RNLs dissociate from the EDS1 heterodimer, resulting in RNL oligomerization and eventually in RNL-mediated immunity (Fig 1) [27]. In summary, EDS1-PAD4-ADR1s and EDS1-SAG101-NRG1s form 2 distinct signaling modules that are essential for many plant immune responses.

## EDS1-PAD4-ADR1s form a convergence hub for PRR- and NLR-mediated immune signaling

*Arabidopsis* ADR1s, EDS1, and PAD4 are not only required for immune signaling initiated by TNLs but also for some plasma membrane (PM)-localized PRRs [5,6]. The PRR RECEPTOR-LIKE PROTEIN 23 (RLP23) and to a lesser extent FLAGELLIN SENSITIVE 2 rely on EDS1, PAD4, and ADR1s to induce full immune outputs [5,6]. RLP23 and its associated adaptor kinase SUPPRESSOR OF BIR 1 form a constitutive complex with EDS1, PAD4, and ADR1s at the PM [5]. This highlights the importance of the EDS1-PAD4-ADR1s signaling module beyond NLR-induced immune signaling and demonstrates why these immune module components are also required for basal resistance [5,7,9,11,19,28], which is considered to be mostly mediated by PRRs. The EDS1-PAD4-ADR1 module thus acts as a convergence point for PRR- and NLR-induced signaling pathways in *Arabidopsis* [29]. This may explain why activation of PRRs and NLRs—although locally separated—result in common immune outputs that only differ in timing and amplitude [1]. However, how pattern-recognition by PRRs activates the EDS1-PAD4-ADR1 module is still under investigation. One hypothesis is that PRR- or PRR co-receptor-associated TNLs may be activated upon ligand binding to the PRR. This could induce confirmational changes or specific dis-/associations leading to TIR-domain enzymatic activity. TIR-dependent NADase activity leads to production of the small signaling molecule(s) initiating the activation of ADR1s by the EDS1-PAD4 heterodimer and initiation of full PRR-triggered responses. The fact that chemical inhibition of TNL enzymatic activity leads to a reduction of some PRR-mediated immune outputs is supporting this hypothesis [30].

## Activated RNLs promote cation influx

The PM localization of *Arabidopsis* RNLs is mediated by the interaction of positively charged residues in the CC_R_ domain with phosphatidylinositol-4-phosphate at the inner leaflet of the PM and is required for RNL cell death function [27,31]. Non-active AtNRG1s (NRG1.1, NRG1.2) were reported to localize not only to the PM, but also to other cellular compartments, including the endoplasmic reticulum and the cytosol, whereas AtADR1s predominantly localize at the PM [9,12,27,32]. Remarkably, the PM localization of (auto-) activated AtNRG1.1 was enhanced compared to non-active wild-type AtNRG1.1 [32]. This suggests that the 2 RNL subfamilies function at the PM, but potentially get activated at different cellular sites. The N-terminal CC_R_ domain of RNLs is structurally similar to the N-terminal cell death inducing domains of the RNL-independent *Arabidopsis* CNL HOPZ-ACTIVATED RESISTANCE 1 (ZAR1), the wheat CNL Sr35 and the mammalian and plant MIXED-LINEAGE KINASE LIKE (MLKL) proteins [19,32–36]. Effector recognition by Sr35 and ZAR1 leads to the formation of a pentameric complex, the resistosome. CNL-resistosomes were shown to function as nonselective Ca^2+^ permeable cation channels that are essential to trigger cell death and immunity, probably by disturbing ion homeostasis and/or activating other cell death/immunity inducing components [35,37,38]. Likewise, members of both RNL subfamilies self-associate and form high-molecular weight complexes upon activation [15,21,27,31]. These are thought to execute cell death specifically at the PM [31,32]. Thus, RNLs may function in a manner similar to ZAR1 and Sr35. Indeed, one of the most remarkable recent findings demonstrated that autoactivated AtNRG1.1 and ectopically active AtADR1 promote nonselective cation influxes in plants and a human cell line [32]. Cation influx induced by ADR1 and autoactivated NRG1.1 ultimately resulted in cell death independent of other plant proteins [32].

Transcriptional analyses revealed that RNLs induce changes that are reminiscent of the transcriptional changes induced by CNLs during pathogen infection [11]. Thus, it appears to be likely that RNLs might use a similar mechanism as, for example, ZAR1 to induce defense responses upon pathogen recognition. Accordingly, RNLs may act as immune and cell death executors downstream of all RNL-dependent sensor NLRs that have no channel activity. How cation influxes following NLR activation specifically result in defense responses, including cell death, remains to be investigated.

## Future directions

Despite their importance for plant innate immunity [5–7,11], the exact molecular mechanism(s) by which RNLs are activated during PRR- and NLR-induced immunity is (are) still unknown. Interestingly, recent work has revealed that PRRs and PRR co-receptors are specifically required for immune responses initiated by helper-dependent and, also to a lesser extent, helper-independent NLRs [2,3]. This suggests that PRR-complexes or downstream-acting kinases might be required for or at least contribute to proper RNL activation. How these proteins could activate or “prime” RNLs is still unclear but given that PRR-complexes and their direct downstream signaling components are kinases, phosphorylation of RNLs could be involved (Fig 1). Future work should address whether RNLs are indeed phosphorylated upon PRR activation and whether phosphorylation is regulating their activity and/or localization.

Another interesting mechanistic question that remains to be solved is whether RNLs trigger cell death and disease resistance by directly disturbing ion homeostasis and if so, how exactly cation influxes induce cell death and transcriptional reprogramming. Upon infection, RNL function and channel formation may be regulated by effector- or PRR-mediated TNL activation, and by PRR kinase activity (Fig 1). This would also be in line with the interdependency and mutual potentiation of ETI and PTI required for full disease resistance [2,3].

Membrane pores formed in mammalian cells during necroptosis, pyroptosis, and apoptosis have been shown to mediate not only cation fluxes but also the release of peptide hormones and pro-inflammatory signals that trigger cell death and activate immunity [39]. Thus, it would be interesting to see whether pathogen-induced RNL (and CNL) activation leading to pore formation and cation influx is also accompanied by the release of host peptides or small molecules to activate defense signaling in neighboring cells (Fig 1).

So far, RNL importance for immunity was only demonstrated for *Arabidopsis* and *Nicotiana* species. Therefore, it will be of great importance to analyze RNL contributions to immunity in other plants that do not belong to the *Brassicaceae* or *Solanaceae* families. In this regard, research on grasses, including important crop plants like rice, corn, or wheat, would be of high interest, since these monocotyledonous plants have lost TNLs and NRG1s [40,41].
